# Physical activity in liver transplant recipients: a large multicenter study

**DOI:** 10.1007/s11739-023-03474-7

**Published:** 2023-11-20

**Authors:** Stefano Gitto, Lucia Golfieri, Filippo Gabrielli, Margherita Falcini, Francesco Sofi, Maria Rosa Tamè, Nicola De Maria, Luca Marzi, Andrea Mega, Giovanna Valente, Alberto Borghi, Paolo Forte, Matteo Cescon, Fabrizio Di Benedetto, Pietro Andreone, Marco Petranelli, Maria Cristina Morelli, Paolo De Simone, Chloe Lau, Laura Stefani, Francesco Vizzutti, Francesca Chiesi, Fabio Marra, MEDITRA Research Group, Giovanni Vitale, Giacomo Bisonti, Filippo Schepis, Erica Villa, Guido Piai, Francesco Giuseppe Foschi, Paolo Magistri, Paola Carrai, Stefania Petruccelli, Marco Corsi, Edoardo Falconi, Roberto Palazzo

**Affiliations:** 1 Internal Medicine and Liver Unit, University Hospital Careggi, Department of Experimental and Clinical Medicine, University of Florence, Largo Brambilla 3, 50134 Florence, Italy; 2 Internal Medicine Unit for the Treatment of Severe Organ Failure, Dipartimento Medico chirurgico delle malattie digestive, epatiche ed endocrino-metaboliche, IRCCS Azienda Ospedaliero-Universitaria di Bologna, Policlinico di Sant’Orsola, Bologna, Italy; 3 Internal and Metabolic Medicine, Department of Medical and Surgical Sciences for Children & Adults, AOU di Modena, University of Modena and Reggio Emilia, Modena, Italy; 4 Unit of Clinical Nutrition, Careggi University Hospital, Department of Experimental and Clinical Medicine, University of Florence, Florence, Italy; 5 Gastroenterology Division, Dipartimento Medico chirurgico delle malattie digestive, epatiche ed endocrino-metaboliche, IRCCS Azienda Ospedaliero-Universitaria di Bologna, Policlinico di Sant’Orsola, Bologna, Italy; 6 Department of Gastroenterology, Azienda Ospedaliero-Universitaria di Modena and University of Modena and Reggio Emilia, Modena, Italy; 7 Division of Gastroenterology, Bolzano Regional Hospital, Bolzano, Italy; 8 Liver Unit for Transplant Management – SATTE, Department of Medical Sciences, AORN Sant’Anna e San Sebastiano, Caserta, Italy; 9 Internal Medicine Unit, Faenza Hospital, Faenza, Italy; 10 Gastroenterology Unit, University Hospital Careggi, Florence, Italy; 11 General Surgery and Transplant Unit, IRCCS Azienda Ospedaliero-Universitaria di Bologna, Policlinico di Sant’Orsola, Bologna, Italy; 12 Hepato-Pancreato-Biliary Surgery and Liver Transplantation Unit, University of Modena and Reggio Emilia, Modena, Italy; 13 Contract Lecturer, Department of Experimental and Clinical Medicine, University of Florence, Florence, Italy; 14 Hepatobiliary Surgery and Liver Transplantation, University of Pisa Medical School Hospital, Pisa, Italy; 15 Centre for Addiction and Mental Health, Toronto, ON Canada; 16 Sports Medicine Center Clinical and Experimental Medicine Department, University of Florence, Florence, Italy; 17 Department of Neuroscience, Psychology, Drug, and Child’s Health (NEUROFARBA), Section of Psychology, University of Florence, Florence, Italy

**Keywords:** Physical activity, Mediterranean diet, Survey, Liver transplant, Cardiovascular risk

## Abstract

**Aim:**

Healthy lifestyle and appropriate diet are of critical importance after liver transplant (LT). We provided an analysis of the main patterns of physical activity and found factors associated with physical activity itself.

**Methods:**

Clinically stable LT recipients were enrolled between June and September 2021. Patients completed a composite questionnaire about physical activity, adherence to Mediterranean Diet (MD), quality of life (QoL), and employment. Correlations were analysed using the Pearson coefficients while different subgroups were compared by *t*-test for independent samples or ANOVAs. Multivariable logistic regression analysis was conducted to find predictors of inactivity.

**Results:**

We enrolled 511 subjects (71% males, mean age 63 ± 10.8 years). One hundred and ninety-three patients reported high level of physical activity, 197 a minimal activity and 121 declared insufficient activity. Among these latter, 29 subjects were totally inactive. Considering the 482 LT recipients performing some kind of physical activity, almost all reported a low-quality, non-structured activity. At multivariate analysis, time from LT (odds ratio 0.94, 95% CI 0.89–0.99, *p* = 0.017), sedentary lifestyle (odds ratio 0.99, 95% CI 0.19–0.81, *p* = 0.012), low adherence to MD (odds ratio 1.22, 95% CI 1.01–1.48, *p* = 0.049), and low level of QoL (physical dimension) (odds ratio 1.13, 95% CI 1.08–1.17, *p* < 0.001), were independently associated with total inactivity.

**Conclusion:**

A large portion of LT recipients report an insufficient level of physical activity or are wholly inactive. Inactivity increases with time from LT and was strongly associated with suboptimal diet and low QoL.

## Introduction

Liver transplant (LT) is an established therapy for subjects with end-stage liver disease and/or hepatocellular carcinoma (HCC) [1]. Both cirrhosis and HCC are well-known risk factors for severe fatigue, protein-energy malnutrition, sarcopenia and physical deconditioning, and all these conditions are associated with a relevant decrease in both quality of life (QoL) and survival [2, 3]. The post-LT normalization of liver function represents the main driver of muscle mass recovery but physical function usually requires a very long time period to return to an adequate level [4, 5]. At the same time, after LT, patients develop metabolic alterations with high incidence of obesity, type II diabetes, hypertension, hyperlipidaemia and metabolic syndrome (MS) [6]. As a consequence, LT recipients show higher rates of cardiovascular (CV) events in comparison to the general population [7]. In particular, up to 58% of LT recipients develop CV risk factors or MS [8], which are mainly due to immunosuppressant drugs and unhealthy lifestyle [9, 10].

Currently, life expectancy of transplanted patients is excellent, with a 90% 1-year and over 70% 5-year survival, favoured by the progressive eradication of HCV infection and the global improvement of surgical and clinical management [1]. Therefore, metabolic and CV complications are expected to progressively rise in the next decades [1]. Similarly to the general population, in the context of LT, a modification of lifestyle represents the first-line approach for both prevention and cure of metabolic disorders as recommended by the European Association for the Study of the Liver (EASL) [1], and exercise has been shown to be effective for the treatment of post-LT MS [8] and for improving the QoL [11]. Here, we provide a detailed analysis of the patterns of physical activity (PA) in a large population of transplanted patients, and identify several factors associated with PA itself.

## Methods

### Study population

In the present cross-sectional, multicenter study, we enrolled clinically stable, adult patients (≥ 18 years old) who underwent LT, followed-up in seven different Italian Hepatology Units. Inclusion criteria were LT performed at least 12 months before enrolment and absence of clinical events during the last 6 months. Exclusion criteria were multiorgan transplant, re-transplant, vascular or biliary complications, systemic disorders (e.g., CV disease, cancer, infection, recurrence of pre-LT liver disease), unstable clinical conditions, or hospital admission in the last 6 months. Human Immunodeficiency Virus infection, deafness, or inability to carry out a telephone interview in full understanding, or holidays in the last 4 weeks represented further exclusion criteria. Patients were enrolled between June 1stand September 30th, 2021.

All patients gave their consent before participating. Trained professional staff scheduled a telephone interview, during which patients answered to the composite questionnaire. Subjects were invited to be alone in a soundless setting. We recorded demographic data including gender, age, transplant date, referral centre, region of residence, education degree, presence of caregiver, alcohol, and tobacco habits. Afterwards, patients completed four questionnaires in an estimated total time of 10–15 min.

### Questionnaires

#### The international physical activity questionnaire

The International Physical Activity Questionnaire (IPAQ) was developed by the World Health Organization in 1998 for the surveillance of PA [12]. Two forms are available. The 27-item long version and the IPAQ-short form (SF) have been validated against accelerometer measurements as a gold standard in 12 countries including Italy [12]. The IPAQ-SF includes 11 items about time spent on walking, vigorous and moderate intensity activity, sedentary activity, and demographic information, including education, and other items concerning comprehension of the questionnaire. Information about PA is reported in minutes per day and/or days per week [13].

The IPAQ-SF investigates three types of activity organized in the three domains. The specific types of activity are walking, moderate-intensity activities and vigorous intensity activities. Frequency (measured in days per week) and duration (time per day) are recorded separately for each activity. The items were structured to provide separate scores on walking, moderate-intensity, and vigorous-intensity activity as well as a combined total score to describe global level of activity.

Another measure of volume of activity can be computed by weighting each type of activity by its energy requirements defined in Metabolic Equivalent Task (MET, multiples of the resting metabolic rate) to yield a score in MET-minutes. A MET-minute is computed by multiplying the MET score by the minutes of activity performed. MET-minute scores are equivalent to kilocalories for a 60-kg person. An average MET score was derived for each type of activity. There are three possible levels of PA suggested for classifying populations which take account of the concept of total PA of all domains. The proposed levels are: (i) inactive; (ii) minimally active; (iii) high active category.

We considered a further sub classification: patients with MET = 0 were considered as totally inactive.

The IPAQ also provides an indicator of sedentary activity, measuring time spent sitting on a typical week expressed in ‘minutes’ (Sitting Total Minutes/week = weekday sitting minutes × 5 weekdays + weekend day sitting minutes × 2 weekend days) [12]. The IPAQ sitting question is an additional indicator variable and is not included as part of any summary score of PA.

We also conducted a qualitative analysis of PA since in the questionnaire patients indicated the prevalent activity. We organized data according to three subgroups: non-structured activity (any bodily movement produced by skeletal muscles that requires energy expenditure), structured (a subset of PA that is planned and repetitive and has as a final or an intermediate objective the improvement or maintenance of physical fitness) and sport activity (it involve PA and exercise but differ in that they also have a set of rules, or goals to train and excel in specific athletic skills) [14, 15].

#### Other questionnaires

Data to calculate the Medi-Lite score were recorded. Medi-Lite represents a validated tool to measure the adherence to MD [16], and consists of nine items about daily consumption of fruit, vegetables, cereals, meat and meat products, dairy products, alcohol, and olive oil, and the weekly intake of legumes and fish [16]. The final score ranges from 0 (low adherence) to 18 (highest adherence).

The QoL was evaluated with the Short Form Health Survey (SF-12), which consists of twelve questions exploring eight health domains to evaluate physical and mental health [17]. We computed two summary scores of physical (Physical Component Summary, PCS-12) and mental (Mental Component Summary, MCS-12) health, using the weighted means of the eight domains.

Finally, we assessed the return to work with both closed and open ad hoc questions, using a specialized employment questionnaire as reported in our recently published study focused on MD [18].

### Statistical analysis

All analyses were conducted with SPSS (version 28.0), after analysing the missing values. Pairwise deletion was used when a case had missing answers.

#### Study population description and analysis of physical activity patterns

Descriptive statistics, such as frequencies, percentages, median, mean [± standard deviation (SD)] were used to describe the sample’s characteristics.

#### Analyses of IPAQ score variables

We investigated the relationships between the IPAQ variables and personal data, life-style patterns, Medi-Lite score and QoL. We computed independent samples *χ*^2^ tests and *t* tests comparing totally inactive (MET = 0) versus active (MET > 0) patients. Specifically, *χ*^2^ tests were used to compare genders, educational levels (primary school, secondary school, high school, and university), place of stay (northern, central, southern Italy), occupation (blue collar, white collar, unemployed/retired), caregiver (yes, no), smoking (yes, no), alcohol habit (none, occasional, continuous). The t tests were used for age, time from LT, Medi-Lite, IPAQ, and the PCS-12 and MCS-12 scores. As measures of effect size, *Cramer’s V* was computed for *χ*^2^ tests.

Mann–Whitney and Kruskal–Wallis non-parametric tests (for two or more groups, respectively) were used to examine the continuous variables such as PA expressed as MET.

#### Multivariate analysis to identify predictors of total inactivity

A multivariable logistic regression analysis was developed to describe and test hypotheses about relationships between the categorical outcome variable (inactive vs. active) and some continuous predictor variables. As indicators of overall model evaluation, we referred to Hosmer–Lemeshow inferential goodness-of-fit test [19] (lower values and non-significance indicate a good fit to the data)and Nagelkerke *R*^2^ [20] (values range from 0 to 1). Statistical significance of individual predictors was tested using the Wald chi-square statistic (*p* < 0.05). The resultant predicted probabilities (odds ratios) can be used to determine if higher or lower probabilities are indeed associated with an event (i.e. inactive patient) given the different levels of the predictor variables (e.g. age). Odds ratios are expressed together with 95% confidence interval.

#### Sample size determination

For observational studies that include logistic regression, a minimum sample size of 500 is conventionally needed to infer the statistics that represent the parameters [21]. The other recommended rules of thumb include the following: *n* = 100 + 50*i*, where *i* refers to number of independent variables in the final [21]. Coherently with the aims of the current study, we hypothesized that at least eight predictors (gender, age, educational level, smoking and alcohol habits, adherence to MD, physical and mental health) will account for the outcome variable. As such, we calculated to enrol at least 500 patients (i.e. 100 + [50 × 8] = 500).

### Ethical approval

The local Independent Ethics Committee approved the study (CEAVC, Tuscany, Italy, approval number 20659) that was developed according to the ethical parameters established in the Declaration of Helsinki (1964) and its later amendments [22].

## Results

### Study population

We administered the composite questionnaire to 511 patients. Most of them were male (71%) and the mean age 63 ± 10.8 years. Enrolled patients showed a homogeneous distribution among the various levels of schooling coming from many Italian areas. Detailed socio-demographic data, clinical information, descriptive of the SF-12 physical (PCS-12) and mental (MCS-12) score, and the Medi-Lite score are reported in Table 1.

**Table 1:** Main demographic, social and life-style patterns

	*N*	%
Gender		
Male	362	70.8
Female	149	29.2
Education		
Primary school	60	11.7
Secondary school	197	38.6
High school	193	37.8
University	61	11.9
Place of residence		
Northern Italy	231	45.2
Central Italy	197	38.6
Southern Italy	83	16.2
Occupation		
Blue collar	111	21.7
White collar	141	27.6
Unemployed/retired	259	50.7
Caregiver		
Yes	152	29.7
No	359	70.3
Smoking		
Yes	116	22.7
No	395	77.3
Alcohol habit		
No	355	69.5
Occasional	124	24.3
Continuous	32	6.3
Hepatology unit		
Bologna	167	32.7
Bolzano	38	7.4
Caserta	32	6.3
Faenza	27	5.3
Firenze	146	28.6
Modena	63	12.3
Pisa	38	7.4

	*M* (SD)	Median (range)
Age (years)	63.1 (10.8)	64 (26–85)
Time from LT (years)	10.69 (7.11)	9 (1–32)
Medi-Lite	10.40 (2.19)	11 (3–16)
PCS-12	47.26 (9.57)	50 (16.60–63.40)
MCS-12	49.33 (9.89)	52 (17.16–67.04)

LT, Liver Transplant; Medi-Lite, adherence to the Mediterranean diet score; PCS-12, Physical health score; MCS-12, Mental health score

In the Table 2, we reported all indicators of IPAQ questionnaires. Among 511 patients, 145 (28.4%) reported at least 1 day per week of vigorous activity (median time per day, 60 min), 390 (76.3%) at least one day per week of moderate activity (median time per day, 60 min), 432 at least 1 day of walking per week (median time per day, 45 min).

**Table 2:** Parameters of the International Physical Activity Questionnaire (IPAQ)

Physical activity	*N*	%	*M* (SD)	Median (range)
Vigorous-intensity activities (days per week)	511	100	1.02 (1.97)	0 (0–7)
Vigorous-intensity activities (minutes per day)	145^a^	28.4	91.03 (92.18)	60 (10–600)
Moderate-intensity activities (days per week)	511	100	3.73 (2.79)	4 (0–7)
Moderate-intensity activities (minutes per day)	390^a^	76.3	94.99 (87.76)	60 (10–600)
Walking (days per week)	511	100	4.20 (2.72)	5 (0–7)
Walking (minutes per day)	432^a^	84.5	63.58 (65.78)	45 (10–600)
Total activity time (MET minutes per week)	511	100	2814.42 (2674.49)	2125 (0–14238)
Sedentary activity (minutes per day)	509^b^	99.6	248.98 (142.71)	200 (60–900)

Metabolic Equivalent Task, MET; patients number, N; mean, M; standard deviation, SD

^a^Number of patients that reported at least 1 day per week of indicated activity

^b^Two patients did not indicate the value (missing data)

**Fig. 1:**
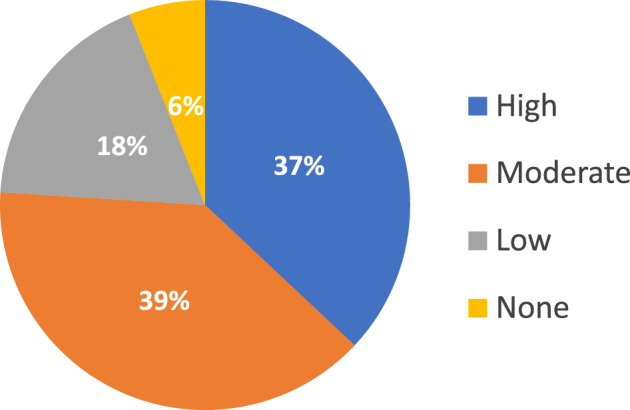
Physical activity levels derived from the International Physical Activity Questionnaire

As reported in Fig. 1, 6% of patients (29/511) resulted to be totally inactive (MET value = 0).

By a qualitative point of view, almost all patients practised non-structured PA (482/511, 94.3%), while in 5.5% (28/511) it was structured, and only 1 patient (0.2%) practised sport activity. Non-structured activity mainly consisted in walking (54.0%) followed by housework (12.7%).

**Fig. 2:**
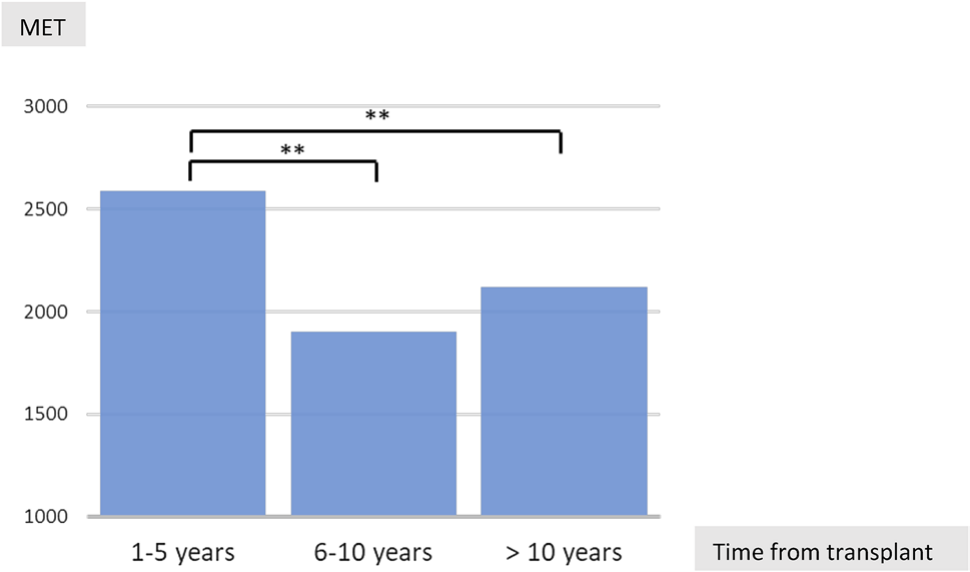
Median of the Total Activity Time by time from transplantation

Total activity Time (expressed in MET) declined according to time from LT (*χ*^2^ [2] = 6.97, *p* = 0.03, Fig. 2). Specifically, the group transplanted 1–5 years before evaluation significantly differed from the 6–10 year group (*z* = − 2.42, *p* = 0.015) and from these transplanted more than 10 year before (*z* = − 2.34, *p* = 0.019), while there were no differences between the latter two groups (*z* = − 0.36, *p* = 0.72).

### Characteristics of the totally inactive patients

The IPAQ parameters were used to compare totally inactive patients (MET = 0, *N* = 29) with all the others (MET > 1, *N* = 482) as showed in Table 3. Inactive patients had a significantly longer time from LT and a higher sedentary activity score of IPAQ. The two groups differed in the Medi-Lite score, indicating that inactive patients had a lower adherence to the Mediterranean diet. Finally, a large discrepancy was found in the PCS-12 score, indicating that inactive patients perceived a lower physical health, while the two groups reported similar levels of mental health.

**Table 3:** Demographic, social, life-style patterns, adherence to adherence to the Mediterranean diet, and physical and mental health by physical activity (inactive vs. active) groups

	Inactive (*N* = 29)	Active (*N* = 482)	*P*-value	Effect size
Gender *N* (%)				
Male	21 (72)	341 (71)	0.85	0.01
Female	8 (28)	141 (29)		
Education *N* (%)				
Primary school	4 (14)	56 (12)	0.62	0.06
Secondary school	14 (48)	183 (38)		
High school	8 (28)	185 (38)		
University	3 (10)	58 (12)		
Place of stay *N* (%)				
Northern Italy	11 (38)	220 (46)	0.67	0.04
Central Italy	12 (41)	185 (38)		
Southern Italy	6 (21)	77 (16)		
Occupation *N* (%)				
Blue collar	4 (14)	107 (22)	0.53	0.05
White collar	8 (28)	133 (28)		
Unemployed/Retired	17 (59)	242 (50)		
Caregiver *N* (%)				
No	10 (35)	142 (30)	0.57	0.03
Yes	19 (65)	340 (70)		
Smoking *N* (%)				
No	19 (66)	376 (78)	0.12	0.07
Yes	10 (34)	106 (22)		
Alcohol habit *N* (%)				
No	17 (59)	338 (70)	0.39	0.06
Occasional	10 (34)	114 (24)		
Continuous	2 (7)	30 (6)		
Mean (SD)				
Age (years)	64.93 (9.88)	62.96 (10.83)	0.34	0.18
Time from LT (years)	10.97 (8.50)	10.50 (6.96)	0.01	0.50
Sedentary activity (minutes per day)	348.97 (200.11)	242.84 (136.45)	< 0.001	0.75
Medi-Lite	9.45 (2.68)	10.46 (2.15)	0.02	− 0.46
PCS-12	34.74 (9.87)	48.02 (9.02)	< 0.001	− 1.47
MCS-12	49.53 (11.58)	49.33 (9.80)	0.91	0.02

Comparisons were made using *χ*^2^ test (categorical variables) and *t*-test (metric variables)

LT, Liver Transplant; Medi-Lite, adherence to the Mediterranean diet score; PCS-12, Physical health score; MCS-12, Mental health score

### Multivariate analysis of total inactivity

Logistic regression was conducted to identify the independent predictors of total inactivity. In inactive patients a longer time had elapsed from LT. In addition, they were more sedentary (higher level of SA-IPAQ) and reported lower adherence to MD and lower QoL-physical health in comparison to the others. The specific weight of each predictor is reported in Table 4.

**Table 4:** Multivariable logistic regression analysis with physical activity (inactive vs. active) as outcome variable

Variable	*β*	SE *β*	Wald’s *χ*^2^	*df*	*p*	Odds ratio (*e*^*β*^)	95% CI (*e*^*β*^)
Time from LT	0.07	0.03	5.70	1	0.017	0.94	0.89–0.99
Sedentary activity	− 0.94	0.37	6.28	1	0.012	0.99	0.19–0.81
Medi-Lite	0.20	0.10	3.89	1	0.049	1.22	1.01–1.48
PCS-12	0.21	0.02	32.69	1	< 0.001	1.13	1.08–1.17

Overall model evaluation: Hosmer and Lemeshow: *χ*^2^ = 6.53, *df* = 8, *p* = 0.59. Nagelkerke *R*^2^ = 0.32

LT, Liver Transplant; Medi-Lite, adherence to the Mediterranean diet score; PCS-12, Physical health score; Variable coding: 0 = inactive, 1 = active

## Discussion

In the general population, PA has been recognized as a critical component of a lifestyle directed to the reduction of CV risk. In fact, low level of PA represents a consolidated risk factor for obesity, type 2 diabetes, CV disease and death [23, 24]. Conversely, regular PA and fitness improve health and decrease the risk of chronic diseases, frailty, disability, falls, and mortality [25, 26]. The positive impact of PA has also been reported in the specific setting of LT where age, metabolic comorbidities, and pharmacologic factors contribute to determine a high CV risk [8]. Remarkably, Kallwitz et al. [8] enrolled 204 LT recipients and analyzed their metabolic patterns with a cross-sectional design study. Authors reported that exercise intensity was the most important protective factor against the development of MS. In fact, low-intensity exercise represented an independent predictor of post-LT MS (OR = 0.690, 95% CI 0.536–0.887). Authors underlined also that a post-transplant adequate care needs a multidisciplinary team that should include dietary, exercise, and behavioural interventions.

Here, we analyzed the patterns of PA in a large cohort of Italian patients homogeneously enrolled across the national territory. We reported that a quarter of patients performed an inadequate level of PA and among them, a consistent subgroup (25%) declared to be totally inactive. It is important to underscore that the strict inclusion and exclusion criteria of the study allowed us to enrol only patients who were clinically stable and without any clinical contraindications to PA. Therefore, considering the transplant population at large, the prevalence of inadequate PA is certainly under-estimated.

To assess the PA, we used the IPAQ-SF [27, 28] which is considered equivalent to the long version [29] although it tends to overestimate the degree of PA [30]. To overcome this intrinsic limitation of the questionnaire, we considered as a reference group the extreme of the score spectrum represented by the totally inactive patients (MET = 0). Interestingly, this group was characterized by a longer time elapsed after LT, in agreement with many previous studies demonstrating how the adherence to the indications provided by the clinicians tend to decrease away from the time of transplant [31–33].

Unfortunately, we have no data about a possible drop in performance status level during the post-transplant follow-up that might explain the registered decrease of PA level. However, performance status is strictly associated to age [34] and, in the present study, older age did not emerge as predictor of impaired PA. In contrast to our data, Kotarska et al. [35] reported that older age negatively correlated to PA. Additionally, authors demonstrated that female gender was significantly associated to low PA levels. The role of age and even more the impact of gender deserve extensive dedicated studies.

As expected, totally inactive patients showed a high score of SA-IPAQ, that is recorded separately from the parameters reporting the degree of PA [13].

Two relevant lines of information provided by the present study are the low levels of QoL-Physical Health and the low adherence to MD in totally inactive patients. Levels of PA were significantly associated to the main determinants of QoL. In particular, post-transplant PA, mobility or total energy expenditure have been previously related with improved QoL [11, 36]. Notably, high-quality PA such as sport practice was associated with improved physical function and QoL for up to 5 years after transplant [37, 38]. Cicognani et al. [37] developed an interesting study comparing 168 sportive transplanted patients (STP), 97 non-sportive transplanted patients (NSTP), and 152 sportive healthy controls (SHC) with the use of SF-36 test. Notably, STP achieved higher scores than NSTP and SHC on Mental Health. Among STP, the effect of quantity of sport activity was noteworthy on both General Health and Role Emotional.

In our study, almost all physically active patients played a low quality and non-structured PA. In the particular context of LT, data are too limited to establish the specific role of structured PA on the patient outcome [39] although some relevant experiences are very promising. Krasnoff et al. [5] developed a randomized clinical trial enrolling 151 LT recipients and comparing combined intervention of exercise and dietary counselling (ExD) *versus* usual care. Although adherence to intervention was suboptimal (37%), patients of ExD group displayed higher rises in VO (2peak) and self-reported general health compared to controls. These data are interesting but should be confirmed with larger studies also because from 2006, the epidemiology of indications for LT has strongly changed [1].

Mosconi et al. [40] reported preliminary results of a multicentre study in which 26 transplanted patients (21 kidney, 5 liver transplanted) completed 12 months of supervised PA. Patients showed an improvement of peak aerobic power, maximum workload in the incremental cycling test and QoL. Moreover, many studies indicated a clear benefit of structured PA in contexts such as older and frail patients [41, 42], which bear similarities with the transplanted patients. Indeed, LT recipients, whenever the general clinical conditions are permissive, should follow a supervised-structured exercise program for improving physical fitness, muscle strength, and functional performance [4]. Interestingly, group-based physical activities may provide a positive incentive to promote a true lifestyle change in the transplant context [43, 44]. The social interaction and teamwork occurring in group-based transplant-related physical activities can deliver significant benefits in terms of self-esteem and confidence [45].

Notably, PA and diet are closely associated since sedentary behaviours are associated with consumption of unhealthy foods in youths [46], in adults [47, 48] and in older subjects [49]. Together with the results of the present study, the available lines of evidence, indicate that lifestyle should be considered as a unique entity comprising diet and nutrition, PA and sports, and social relationships. Consequently, all the actions directed to improve lifestyle must be comprehensive and complete.

Specific consensus about PA target levels for LT recipients are not available, but it seems reasonable to follow the United States population guidelines, which consist of 150 min/week of moderate-to-vigorous activity, along with resistance exercise training for 15–20 min twice weekly [50]. Dunn et al. [51] underlined the relevance of a continuous PA counseling during the post-transplant follow-up visits. Transplant hepatologist should encourage group or recreational sporting activity whenever clinically possible [38, 44]. In addition, it will be important to implement the use of new technologies. In this respect, Hickman et al. [52] examined the feasibility of a 12-week telehealth-delivered lifestyle intervention for LT recipients, reporting that a cardioprotective engagement is feasible and useful to enhance the post-transplant metabolic wellness.

Some limitations of this study should be acknowledged. First, it is based on self-reported assessments and not on objective measurements. Moreover, the study has a cross-sectional design and thus, causality between parameters, specifically between inactivity and low adherence to MD and low QoL, could not be established. As further limitations, we have no data about CV events that represent main outcomes for diet and PA interventions that are topics of the present study. Moreover, we did not register anthropometric parameters, equally very important in the field of PA and nutrition. Seeing the noteworthy role of immunosuppressive therapy in conditioning all the main metabolic patterns, the lack of data about these drugs certainly represents another limitation of the present study.

In conclusion, we demonstrated that a large part of clinically stable LT recipients declares an inadequate PA or are completely inactive. Inactivity significantly increases with time elapsed from LT and it was closely associated with unhealthy diet and poor QoL. Among patients who perform PA, only a small minority practice high-quality activity. The transplant community should increase its efforts to promote post-transplant, high-quality, lifelong PA.

## Data Availability

Not applicable.
